# A comparative human rights analysis of laws and policies for adolescent contraception in Uganda and Kenya

**DOI:** 10.1186/s12978-021-01303-8

**Published:** 2022-02-07

**Authors:** Katrina Perehudoff, Denis Kibira, Elke Wuyts, Carles Pericas, Joyce Omwoha, Hendrika A. van den Ham, Aukje K. Mantel-Teeuwisse, Kristien Michielsen

**Affiliations:** 1 International Centre for Reproductive Health, Department of Public Health and Primary Care, Ghent University, C. Heymanslaan 10, 9000 Gent, Belgium; 2 Amsterdam Law School, University of Amsterdam, Amsterdam, The Netherlands; 3 Academic Network on Sexual and Reproductive Health and Rights Policy (ANSER), Ghent University, C. Heymanslaan 10, 9000 Gent, Belgium; 4 Coalition for Health Promotion and Social Development (HEPS-Uganda), Plot 351A, Balintuma Road, Namirembe Hill, Kampala, Uganda; 5 Department of Journalism and Media Studies, Faculty of Social Sciences and Technology, Technical University of Kenya, P.O. Box 52428, Nairobi, Kenya; 6 Division of Pharmacoepidemiology and Clinical Pharmacology, Utrecht Institute for Pharmaceutical Sciences (UIPS), Utrecht University, Universiteitsweg 99, 3584 CG Utrecht, The Netherlands

**Keywords:** Adolescent pregnancy, Contraception, Human rights, Health policy, Uganda, Kenya

## Abstract

**Background:**

Improving access to adolescent contraception information and services is essential to reduce unplanned adolescent pregnancies and maternal mortality in Uganda and Kenya, and attain the SDGs on health and gender equality. This research studies to what degree national laws and policies for adolescent contraception in Uganda and Kenya are consistent with WHO standards and human rights law.

**Methods:**

This is a comparative content analysis of law and policy documents in force between 2010 and 2018 governing adolescent (age 10–19 years) contraception. Between and within country differences were analysed using WHO’s guidelines “Ensuring human rights in the provision of contraceptive information and services”.

**Results:**

Of the 93 laws and policies screened, 26 documents were included (13 policies in Uganda, 13 policies in Kenya). Ugandan policies include a median of 1 WHO recommendation for adolescent contraception per policy (range 0–4) that most frequently concerns contraception accessibility. Ugandan policies have 6/9 WHO recommendations (14/24 sub-recommendations) and miss entirely WHO’s recommendations for adolescent contraception availability, quality, and accountability. On the other hand, most Kenyan policies consistently address multiple WHO recommendations (median 2 recommendations/policy, range 0–6), most frequently for contraception availability and accessibility for adolescents. Kenyan policies cover 8/9 WHO recommendations (16/24 sub-recommendations) except for accountability.

**Conclusions:**

The current policy landscapes for adolescent contraception in Uganda and Kenya include important references to human rights and evidence-based practice (in WHO’s recommendations); however, there is still room for improvement. Aligning national laws and policies with WHO’s recommendations on contraceptive information and services for adolescents may support interventions to improve health outcomes, provided these frameworks are effectively implemented.

**Supplementary Information:**

The online version contains supplementary material available at 10.1186/s12978-021-01303-8.

## Plain language summary

The unmet need for contraception among adolescents is high in Uganda and Kenya, and has many negative consequences, including unwanted pregnancy, exposure to unsafe abortion, and maternal morbidity and mortality. National laws and policies play an important role in determining adolescents’ access to contraception. For example, national laws and policies can shape the government programs that provide (or withhold) contraception, and the social norms influencing adolescents’ access to contraception. Therefore, this research compares national laws and policies that determine access to contraception services and information for adolescents in Uganda and Kenya with WHO’s recommendations for access to contraception.

This is an analysis of the content of Ugandan and Kenyan laws and policies in force between 2010 and 2018. The content of these documents was analyzed using WHO’s nine recommendations for how contraception information and services should be provided: non-discrimination, availability, accessibility, acceptability, quality, informed decision-making, confidentiality, participation, accountability.

Ninety-three documents were screened and 26 documents were included in the analysis: 13 policies from Uganda and 13 policies from Kenya. On average, Ugandan policies include one WHO recommendation for adolescent contraception per policy and Kenyan policies include two WHO recommendations. This recommendation most frequently mentioned in all policies is the accessibility of contraception (for example, for adolescents living remotely, integrated in adolescent HIV or pre-/post-natal care, etc.). Together, all Ugandan policies mentioned 6/9 WHO recommendations whereas all Kenyan policies cover 8/9 WHO recommendations.

In conclusion, Ugandan and Kenyan policies are consistent with many of WHO’s recommendations for access to contraception, however, there is still room for improvement.

**Supplementary Information:**

The online version contains supplementary material available at 10.1186/s12978-021-01303-8.

## Background

The 2030 Agenda for Sustainable Development seeks to improve gender equality (Sustainable Development Goal (SDG) 5) and reduce maternal mortality (SDG 3), which claims the lives of 289,000 women annually [1]. Worldwide, pregnancy-related causes are the leading cause of death among women aged 15–24 and this is mostly in low- and middle-income countries [2]. Improving access to contraceptive information and services is essential to reduce maternal mortality [3, 4].

Achieving the SDG goals requires adopting supportive national sexual and reproductive health (SRH) laws and policies and repealing regressive legal rules, for example restrictions on contraception and abortion services. Recent changes in global and national SRH policies illustrate that the policy making process is dynamic and complex, sometimes leading to public health decisions unsupported by evidence and/or human rights by governments, public health officials, health providers, and/or other SRH actors [5]. In the case of access to contraception for adolescents, one school of thought favours restricting access based on the notion of traditional morals, values, and culture; while another school of thought promotes contraceptive access based on the best medical evidence and human rights law [6]. In this paper we refer to adolescents as people aged 10–19 years as defined by the World Health Organisation (WHO).

Both Uganda and Kenya have a high maternal mortality rate (336 and 362 per 100,000 live births, respectively), a low prevalence of modern contraceptive use among adolescents aged 15–19 years in union (7% and 20% respectively,) and high fertility rates among adolescents aged 15–19 years ((132/1000 and 96/1000 women, respectively), respectively) [7, 8]. During the tenure of the Millennium Development Goals (MDGs), both governments modestly improved contraceptive uptake with the use of modern contraceptives among married women increasing in Uganda from 18 (2001) to 35% (2016) and in Kenya from 31.5 (2003) to 53% (2014) and adopted laws and policies promoting adolescent contraception [9, 10]. These achievements were realised against the backdrop of restrictive gender norms, some political opposition often from religious communities, and negative media messages about contraception [11]. The Ugandan government’s decision to endorse the 2018 National Sexuality Education Framework made significant headlines by effectively abolishing comprehensive sexuality education for adolescents including contraception in favour of a values-based approach that favours abstinence promotion [12, 13].

National law and policy are important tools that shape legal obligations, government programmes, social norms, and the potential to hold governments accountable for fulfilling girls’ and women’s health rights [1]. The East African Community (EAC), a regional intergovernmental organisation including Kenya and Uganda, adopted the HIV & AIDS Prevention and Management Act in 2012 that requires EAC States to take a rights-based approach to ensuring adolescents have access to SRH information and education, including about contraception [14].

It is unclear to what extent the national laws and policies in Kenya and Uganda uphold contraceptive availability, accessibility, acceptability, quality, freedom from discrimination, and other standards in the WHO’s guidance document called “Ensuring human rights in the provision of contraceptive information and services” [15]. WHO’s guidelines offer a global standard of evidence-informed and human rights-based recommendations for adolescent contraception; they have been used to analyse domestic law and policy in South Africa, the Philippines, and Paraguay, as well as to guide country-level action [16–20]. Failing to address the norms underlying law, policy, and public health practice may be why some micro-level interventions and programmes are unable to deliver improved adolescent SRH outcomes [21]. Laws and policies that protect and promote adolescents’ access to safe and affordable contraception of assured quality in a non-discriminatory manner can create a supportive environment for the enjoyment of their rights, poverty reduction, and sustainable development.

## Methods

This article aims to assess to what degree national laws and policies for adolescent contraception (2010–2018) in Uganda and Kenya are consistent with the global standards in the WHO’s guidance. This study is a comparative content analysis of legislation and policy documents governing any aspect of adolescent contraception at the national level in Uganda and Kenya. As has been described in previous studies, we defined policy as principles or strategies for a plan of action designed to achieve a particular set of goals, including through guidelines, plans, and standards [22]. During the period 2010–2018 accelerated global efforts were undertaken to improve girls’ and women’s health, starting with the United Nations (UN) Secretary-General’s Commission on Life-Saving Commodities (UNCOLSC, 2010) for Women and Children was set up [23] and in 2015, the UN *Global Strategy for Women’s and Children’s and Adolescents’ Health* (UNGS WCAH, 2016–2030) was published [24]. A cut-off of 2018 was selected in order to catch the initial implementation of the UNGS WCAH.

### Data collection

An online search (conducted in May 2019 and repeated in March 2021) identified relevant laws and policies that were in force between 2010 to 2018 through national government websites and legal databases (i.e. Uganda Legal Information Institute https://ulii.org/consol_leglist/consolidated_legislation, Kenya Law http://kenyalaw.org/kl/index.php?id=400, Kenyan Health Guidelines, Standards & Policies Portal http://guidelines.health.go.ke, Ugandan Ministry of Health Knowledge Management Portal http://library.health.go.ug, International Labour Organisation NATLEX), reference lists in relevant academic commentary and publications, a Google search using the search syntax “((adolescent OR sexual OR Reproductive) health) AND (law or policy OR policies) AND Uganda/Kenya”, and through crowdsourcing documents from our network.

**Fig. 1:**
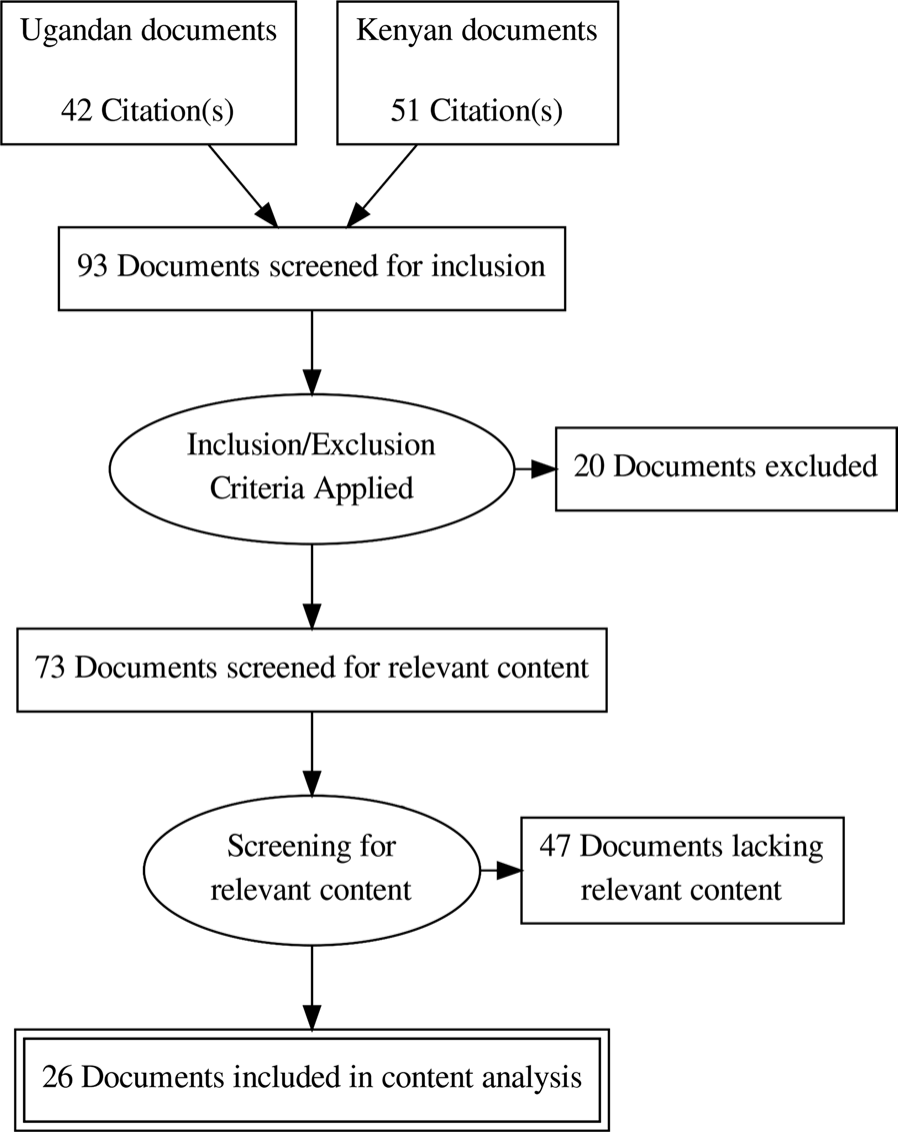
Flow diagram of the selection and inclusion of laws and policies in this study

Documents were selected for inclusion in three stages (See flow diagram in Fig. 1). First, legal, strategic, and policy documents addressing subjects related to adolescent health, SRH, and/or contraception were collected for further screening (See Additional file 1 for a complete list of these documents). Second, we applied the following two inclusion criteria: (1) document is legal, strategic, or policy-related; and (2) the document was in force between 2010 and 2018. We determined whether the document was in force by the term stated explicitly in the document or in a superseding document, the status of the document on a government website, or, in the absence of other available information, by assuming that policy documents have a lifespan of 10 years. Documents were excluded if they did not meet the inclusion criteria or if no full text was available. Third, we scanned the full text to identify any explicit content related to the recommendations in WHO’s guidance document called “Ensuring human rights in the provision of contraceptive information and services” (‘WHO recommendations’). This resulted in a short list of documents that underwent content analysis (see Table 1) [15]. Finally, local policy experts (DK, DKA, JO, TSG) verified whether the shortlist of documents was accurate, complete, and up-to-date, and suggested other documents for consideration. Experts were co-authors (DK, Executive Director of a Ugandan health policy NGO; JO, Kenyan academic expert in gender policy) and project advisors (DKA, TSG—both are health lawyers with track records representing Ugandan and Kenyan NGOs (respectively) with a strong focus on SRH and rights) who have extensively engaged with SRH policies in their respective countries.

**Table 1:** Ugandan and Kenyan laws and policies (in force between 2010 and 2018) included in content analysis

Document title	Date of publication	Status*	Acronym in Fig. 1
*Legal and policy documents from Uganda*			
Uganda Family Planning Cost Implementation Plan 2015–2020 (Ministry of Health)	2015	Current	UFPCIP
National HIV/AIDS Strategic Plan 2015–2020 and Priority Action Plan (Uganda AIDS Commission/Republic of Uganda)	2015	Current	NASP
Reducing Morbidity and Mortality from Unsafe Abortions Standards and Guidelines (Ministry of Health)	2015		RMMUA
National Condom Programming Strategy 2013–2015 (Ministry of Health)	2013		NCPS
Adolescent Health Policy Guidelines and Service Standards (Ministry of Health)	2012	Current	AHPGSS
National Strategic Plan for HIV/AIDS 2011–2015 (Republic of Uganda)	2011		NSPHA
National HIV Prevention Strategy for Uganda 2011–2015 (Republic of Uganda)	2011		NHPS
National HIV Testing Services Policy and Implementation Guidelines Uganda (Ministry of Health)	2010		NHTSPIG
Reproductive Health Commodity Security Strategic Plan, 2009/10– 2013/14 (Ministry of Public Health and Sanitation, Ministry of Medical Services)	2009		RHCSSP
Roadmap for Accelerating the Reduction of Maternal and Neonatal Mortality and Morbidity in Uganda (Republic of Uganda)	2007		RMNMM
National HIV/AIDS Strategic Plan 2007–2012 (Republic of Uganda)	2007		NHSP
National Policy Guidelines and Service Standards for SRH and Rights (Ministry of Health)	2006		NPGSS
National Adolescent Health Policy (Ministry of Health)	2004		NAHP
*Legal and policy documents from Kenya*			
National Family Planning Costed Implementation Plan 2017–2020 (Ministry of Health)	2017	Current	KFPCIP
National Guidelines for Provision of Adolescent and Youth Friendly (AYF) Services in Kenya (Ministry of Health)	2016	Current^	NGPAYS
National Adolescent Sexual and Reproductive Health (SRH) Policy 2015 (Ministry of Health)	2015	Current^	ASRHP
Population Policy for National Development 2012–2030 (Ministry of State Planning, National Development, and Vision 2030)	2012–2030	Current	PPND
National Communication Strategy for Community Health Services 2012–2017 (Ministry of Public Health & Sanitation)	2012	Current*	NCSCHS
Towards the Elimination of Mother to Child Transmission (eMTCT) of HIV and Keeping Mothers Alive- Strategic Framework 2012–2015 (Ministry of Health)	2012	Current*	MTCTSF
Reproductive Health Communication Strategy 2010–2012 (Ministry of Public Health & Sanitation, and Ministry of Medical Services)	2010		RHCS
National Road Map for Accelerating the Attainment of the MDGs Related to Maternal Health and Newborn Health in Kenya (Ministry of Public Health & Sanitation)	2010	Current*	NRMMNH
National Family Planning Guidelines for Service Providers (Ministry of Public Health & Sanitation)	2010		NFPGSP
National Reproductive Health Strategy 2009–2015 (Ministry of Public Health & Sanitation, and Ministry of Medical Services)	2009	Current*	NRHS
Strategy for Improving the Uptake of Long-acting and Permanent Methods of Contraception in the Family Planning Program 2008–2010 (Ministry of Public Health & Sanitation)	2008		SULAR
National Reproductive Health Policy (Ministry of Health)	2007		NRHP
Adolescent Reproductive Health and Development Policy 2005–2015 Plan of Action (Ministry of Planning & Ministry of Health)	2005	Current*	ARHDP

A list of all documents reviewed for this study can be found in Additional file 1

*AIDS* acquired immunodeficiency syndrome, *AYF* adolescent- and youth-friendly, *HIV*  human immunodeficiency virus, *SRH*  sexual and reproductive health

*In these cases the policy’s explicit timeframe had lapsed yet the Ministry of Health still listed the document as ‘current’

^When not explicitly stated in the policy, we assume policies have a 10-year lifespan

### Data analysis

Three researchers (EW, CP, KP) were responsible for coding the documents and the data extraction. A pre-defined coding strategy and data extraction sheet was used, which was pre-tested by these researchers on legal and policy documents from South Africa.

During analysis the included documents were screened for content relating to adolescents, and excerpts were classified by two independent researchers (a medical doctor (CP) and, a health scientist (EW) both trained in health policy, and a health scientist trained in law (KP)) using WHO’s nine recommendations and 24 sub-recommendations, which served as a coding matrix. See Table 2 for an overview of these recommendations.

**Table 2:** Overview of the current Ugandan and Kenyan policy landscape for adolescent contraception according to the nine WHO recommendations

WHO Recommendation		Uganda		Kenya	
		AC	C	AC	C
1. Non-discrimination in provision of contraceptive information and services					
1.1 Recommend that access to comprehensive contraceptive information and services be provided equally to everyone voluntarily, free of discrimination, coercion or violence (based on individual choice)		✔	✔	✔	
1.2 Recommend that laws and policies support programmes to ensure that comprehensive contraceptive information and services are provided to all segments of the population. Special attention should be given to disadvantaged and marginalized populations in their access to these services		✔	✔	✔	
2. Availability of contraceptive information and services					
2.1 Recommend integration of contraceptive commodities, supplies and equipment, covering a range of methods, including emergency contraception, within the essential medicine supply chain to increase availability. Invest in strengthening the supply chain where necessary in order to help ensure availability			✔	✔	✔
3. Accessibility of contraceptive information and services					
3.1 Recommend the provision of scientifically accurate and comprehensive sexuality education programmes within and outside of schools that include information on contraceptive use and acquisition		✔	✔	✔	✔
3.2 Recommend eliminating financial barriers to contraceptive use by marginalized populations including adolescents and the poor, and make contraceptives affordable to all			✔	✔	✔
3.3 Recommend interventions to improve access to comprehensive contraceptive information and services for users and potential users with difficulties in accessing services (e.g. rural residents, urban poor, adolescents)		✔	✔	✔	✔
3.4 Recommend special efforts be made to provide comprehensive contraceptive information and services to displaced populations, those in crisis settings, and survivors of sexual violence, who particularly need access to emergency contraception			✔	✔	✔
3.5 Recommend that contraceptive information and services, as a part of SRH services, be offered within HIV testing, treatment and care provided in the health-care setting		✔	✔	✔	✔
3.6 Recommend that comprehensive contraceptive information and services be provided during antenatal and postpartum care		✔	✔	✔	✔
3.7 Recommend that comprehensive contraceptive information and services be routinely integrated with abortion and post-abortion care		✔	✔	✔	✔
3.8 Recommend that mobile outreach services be used to improve access to contraceptive information and services for populations who face geographical barriers to access			✔		✔
3.9 Recommend elimination of third-party authorization requirements, including spousal authorization for individuals/women accessing contraceptive and related information and services		✔	✔		
3.10 Recommend provision of SRH services, including contraceptive information and services, for adolescents without mandatory parental and guardian authorization/notification, in order to meet the educational and service needs of adolescents		✔	✔		
4. Acceptability of contraceptive information and services					
4.1 Recommend gender-sensitive counselling and educational interventions on family planning and contraceptives that are based on accurate information, that include skills building (i.e. communications and negotiations), and that are tailored to meet communities’ and individuals’ specific needs		✔	✔	✔	✔
4.2 Recommend that follow-up services for management of contraceptive side-effects be prioritized as an essential component of all contraceptive service delivery. Recommend that appropriate referrals for methods not available on site be offered and available			✔		✔
5. Quality of contraceptive information and services					
5.1 Recommend that quality assurance processes, including medical standards of care and client feedback, be incorporated routinely into contraceptive programmes			✔		✔
5.2 Recommend that provision of LARC methods should include insertion and removal services, and counselling on side-effects, in the same locality					✔
5.3 Recommend ongoing competency-based training and supervision of health-care personnel on the delivery of contraceptive education, information and services. Competency-based training should be provided according to existing WHO guidelines			✔	✔	✔
6. Informed decision-making					
6.1 Recommend the offer of evidence-based, comprehensive contraceptive information, education and counselling to ensure informed choice		✔	✔	✔	✔
6.2 Recommend every individual is ensured the opportunity to make an informed choice for their own use of modern contraception (including a range of emergency, short-acting, long-acting and permanent methods) without discrimination		✔	✔	✔	✔
7. Privacy and confidentiality					
7.1 Recommend that privacy of individuals is respected throughout the provision of contraceptive information and services, including confidentiality of medical and other personal information		✔	✔	✔	✔
8. Participation					
8.1 Recommend that communities, particularly people directly affected, have the opportunity to be meaningfully engaged in all aspects of contraceptive programme and policy design, implementation and monitoring		✔	✔	✔	✔
9. Accountability					
9.1 Recommend that effective accountability mechanisms are in place and are accessible in the delivery of contraceptive information and services, including monitoring and evaluation, and remedies and redress, at the individual and systems levels			✔		✔
9.2 Recommended that evaluation and monitoring of all programmes to ensure the highest quality of services and respect for human rights must occur Recommend that, in settings where PBF occurs, a system of checks and balances should be in place, including assurance of non-coercion and protection of human rights. If PBF occurs, research should be conducted to evaluate its effectiveness and its impact on clients in terms of increasing availability			✔		✔
Total	Recommendations for adolescent contraception (AC)	6		8	
	Sub-recommendations for adolescent contraception (AC)	14		16	

*AC*  adolescent contraception, *C*  contraception in general, *LARC*  long-acting reversible contraception, *PBS*  performance-based financing

Researchers used the coding matrix that scored the content based on references to: (1) adolescent contraception, or (2) contraception in general (not specifically related to adolescents). Discrepancies were deliberated until consensus was reached. The coding results were reviewed by two experts (DK, JO) with first-hand knowledge of the local policy context.

Three researchers (KP, CP, EW) investigated between and within country trends. The between-country analysis examined the similarities and differences in the overall legal and policy frameworks that are currently in force. The within-country descriptive analysis examined the evolution in the adolescent-related content of relevant laws and policies in force in both countries between 2000 and 2018.

A stakeholder validation meeting was held at the Ugandan Ministry of Health (MoH) on December 6th, 2019 to discuss the preliminary findings. Due to the Coronavirus pandemic a comparable stakeholder validation meeting in Kenya was not possible.

## Results

Ninety-three documents were screened for inclusion. Of those, sixty-seven documents were excluded from content analysis because they were never adopted (n = 4); retired or superseded before 2010 (n = 8); clinical in nature (n = 3); no full text was available (n = 5); or did not include content related to WHO’s recommendations for contraception information and services (n = 47). This resulted in twenty-six documents being included in content analysis (see flow diagram in Fig. 1).

Of the included documents, 13 were from Uganda and 13 were from Kenya (Table 1).

### Adolescent contraception policy landscape

National laws and policies currently in force in Uganda include six of the nine WHO recommendations (and 14/24 sub-recommendations) and in Kenya they include eight of the nine WHO recommendations (and 16/24 sub-recommendations) for adolescent contraception (Table 2).

**Fig. 2:**
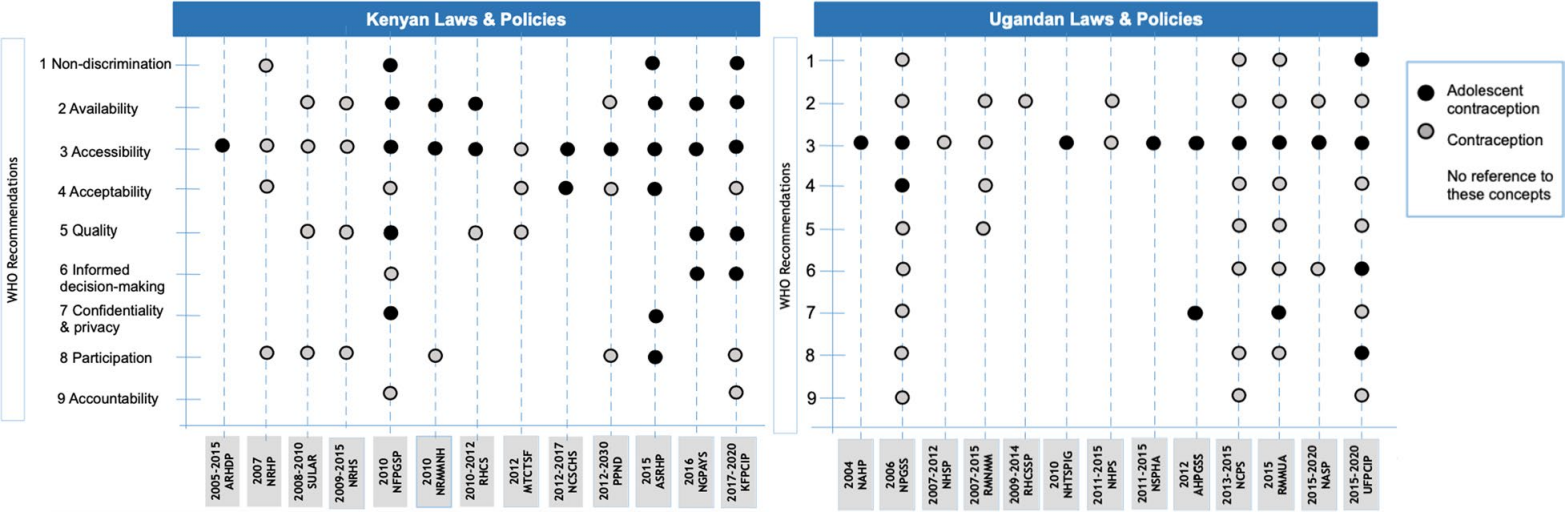
Content of Ugandan and Kenyan policies in force between 2010 and 2018 addressing adolescent contraception

National laws and policies addressed a median of one recommendation for adolescent contraception (range 0–4 recommendations) in Uganda and two recommendations for adolescent contraception (range 0–6 recommendations) in Kenya. Recommendations were concentrated in relatively few policies: Documents with four or more WHO recommendations were found in one Ugandan policy and four Kenyan policies (see Fig. 2). Below we present evidence for each recommendation related to adolescent contraception from the legal and policy documents currently in force in Uganda and Kenya (see Tables 1 and 2, and Fig. 2).

1. Non-discrimination

Non-discrimination refers to equal access that is free from discrimination, coercion or violence (recommendation 1.1(R1.1)). Uganda’s *Family Planning Cost Implementation Plan (2015–2020)* seeks to increase age-appropriate knowledge and access to family planning amongst adolescents (ages 10–24 years), including to disadvantaged or marginalised populations through AYF corners and extended service delivery hours outside school hours (R1.2).

Kenya’s *National Adolescent SRH Policy (2015)* foresees the provision of accurate information and services to prevent the early/unintended pregnancy. The *Family Planning Cost Implementation Plan (2017–2020)* focuses on social behavioural change communication strategy/policy, addressing myths and misconceptions about modern contraceptives; and providing information and services to special needs groups (ex. adolescents), and age-appropriate family planning information (R1.1, R1.2).

2. Availability

Availability requires that contraceptive commodities, supplies and equipment are integrated in the national essential medicine supply chain (R2.1). This was not addressed in relation to adolescent contraception in Uganda’s policies currently in force.

Recommendation 2.1 was the most well covered domain in Kenyan policies. Availability is a focal point in the *National Guidelines for Provision of Adolescent and Youth Friendly (AYF) Services (2016)* (ex. a continuous supply of essential medical commodities via AYF service delivery points).

3. Accessibility

Accessibility concerns barriers to access to contraceptive information and services including information and education, financing, geography, conflict and violence, integration in SRH, maternal, and/or abortion care, and third-party authorisation.

The *Uganda Family Planning Cost Implementation Plan (2015–2020)* seeks to increase adolescent’s knowledge and empowerment to use family planning services and avoid teenage pregnancy through peer educators, print and online media targeting adolescents, and ‘edutainment’ community events. These measures neither mention the scientific basis nor comprehensiveness of information (required for R3.1). This policy also addresses the contraceptive needs of those with difficulty accessing services (R3.3) through AYF services, corners, and delivery hours (ex. outside school hours). Uganda’s *Adolescent Health Policy Guidelines and Service Standards (2012)* require that adolescents have access to a range of SRH services, such as post abortion care and management (R3.7, R3.5 R3.6). Ugandan policies do not mention the elimination of financial barriers for adolescents (R3.2), provision to displaced adolescents, those in crisis settings, or survivors of violence (R3.4), nor the use of mobile outreach services to overcome geographic barriers to access (R3.8). The elimination of third party / parental authorisation in relation to adolescent contraception was not addressed in the policies currently in force (R3.9, R3.10).

Kenya’s *National Family Planning Cost Implementation Plan (2017–2020)* addresses the contraceptive needs of those with difficulty accessing services (R3.3) through training and supporting peer educators, and through age-appropriate family planning information, and a provider-led approach to create demand for services among groups with special needs.

The *National Guidelines for Provision of AYF Services (2016)* define the essential package of services and information that must be available at AYF service points. The essential package integrates contraception counselling and provision of full range of contraceptive methods, including long-acting reversible contraceptive (LARC) methods, with other forms of SRH care (ex. HIV counselling, testing, and treatment) (R3.5), antenatal and postpartum care including pregnancy testing (R3.6), post abortion care (R3.7), and sexual and gender-based violence counselling, services and referrals (R3.4). The policy’s four service delivery models aim to better reach adolescents through (1) community-based initiatives in non-medical settings to reach first time parents, fathers, and young mothers with limited mobility, (2) clinical settings, (3) school settings, and (4) virtually through digital platforms (R3.3). It also prescribes the Minimum Initial Service Package for Reproductive Health (‘essential package’), which is a list of priority interventions designed to reduce mortality, morbidity, and disability among populations affected by crises. The Essential Package should ensure access to a broad mix of ‘free’ family planning methods (R3.2, R3.3, R3.4) that are integrated with HIV prevention and treatment, and maternal care (R3.5, R3.6).

The *National Adolescent SRH Policy (2015)* requires enhancing the provision of high-quality post-abortion services to adolescents, which includes contraceptives (R3.7). No legal documents in Kenya address the elimination of financial barriers for adolescents (R3.2), mobile outreach services (R3.8), nor third-party / parental authorisation (R3.9, R3.10).

4. Acceptability

Acceptability refers to gender-sensitive counselling and educational interventions (R4.1) and follow-up services for the management of contraceptive side-effects (R4.2).

These recommendations were not addressed in Uganda’s policies currently in force. Kenya’s *Adolescent SRH Policy (2015)* takes a gender-sensitive approach to interventions (R4.1) by promoting male involvement in the prevention of early and unintended pregnancy. Neither Ugandan nor Kenyan policies include R4.2 in relation to adolescents.

5. Quality

Quality refers to quality assurance processes for standards of care and client feedback (R5.1), services for the provision and follow-up of LARC methods (R5.2), and competency-based training and supervision of healthcare personnel (R5.3).

Current policies in Uganda and Kenya do not refer to recommendations 5.1–5.2 for adolescent contraception. Kenya’s *Family Planning Cost Implementation Plan (2017–2020)* refers to competency-based training and supervision of healthcare personnel (R5.3) for the practical application of family planning skills, adolescent-friendly service approaches, and internships for graduates to enhance their family planning service provision.

6. Informed decision-making

Informed decision-making requires evidence-based, comprehensive information, education and counselling (R6.1) and the assurance that every individual has the opportunity to make an informed choice about their use of modern contraceptives (R6.2).

Uganda’s *Family Planning Cost Implementation Plan 2015–2020* references provisions for empowering adolescents (age 10–24 years) to use family planning services and AYF information channels with the objective of empowering youth to avoid teenage pregnancy. However, evidence-based information about a range of methods for comprehensive, informed choices is not addressed in relation to adolescent contraception (R6.1), nor is the use of modern contraception without discrimination (R6.2).

Kenya’s *Family Planning Cost Implementation Plan 2017–2020* promotes behavioural change to address myths and misconceptions to improve acceptance and continued use of family planning with a special focus on age-appropriate information, access, and the use of family planning among adolescents (ages 10–24). This policy alludes to the importance of contraceptive information, counselling, and education to ensure informed choice (R6.1) about own use of modern contraception without discrimination (R6.2) in relation to adolescents. Kenya’s *National Guidelines for Provision of AYF Services* (2016) requires that contraception counselling and the full range of contraceptive methods are in essential packages of AYF services (R6.2).

7. Privacy and confidentiality

Privacy and confidentiality refer to the respect of an individual’s privacy through the provision of contraception and confidentiality of medical and personal information (R7.1). Uganda’s *2012 Adolescent Health Policy Guidelines & Service Standards* emphasise the offer of confidential counselling and information and provision of family planning to adolescents. Kenya’s *National Adolescent SRH Policy 2015* requires that all adolescent SRH services in this policy (including contraception) should be offered in a non-judgmental and confidential way.

8. Participation

Participation encompasses the involvement of people directly affected by policies to have the opportunity to be meaningfully engaged in all aspects of programme and policy design (R8.1).

Uganda’s *Family Planning Cost Implementation Plan (2015–2020)* specifically seeks to align with other national policies and strategies and strengthen family planning policy environment including through the participation of women, adolescents, and marginalised and excluded groups.

Kenya’s *National Adolescent SRH Policy 2015* seeks to strengthen ‘community involvement’ to prevent early and unintended pregnancies. This policy seeks to involve adolescents in the planning, implementation, monitoring and evaluation of adolescent SRH programmes.

9. Accountability

Accountability requires that effective and accessible mechanisms exist for the delivery of contraceptive information and services to adolescents (R9.1) as well as programme evaluation and monitoring to ensure the highest quality services and respect of human rights (R9.2). Neither Uganda’s nor Kenya’s documents address these recommendations towards adolescents.

## Discussion

These results illustrate that the current Ugandan and Kenyan policy landscapes for adolescent contraception include important references to human rights and evidence-based practice (in WHO’s recommendations); however, there is still room for improvement. Ugandan policies include infrequent (one-off) recommendations for 6/9 WHO recommendations, commonly in relation to contraception accessibility for adolescents. However, Ugandan policies miss entirely WHO’s recommendations for adolescent contraception availability, quality, and accountability. On the other hand, most Kenyan policies consistently address multiple WHO recommendations, most frequently for contraception availability and accessibility for adolescents. Kenyan policies cover 8/9 WHO recommendations except for accountability. In light of the key policy gaps identified in this study, we provide specific recommendations based on WHO’s guidance and the inter-country comparison for both Uganda and Kenya (see Table 3).

**Table 3:** Key areas of improvement for Ugandan and Kenyan policy frameworks, based on WHO’s recommendations

WHO 9 main recommendations	Priority improvements to current policies in Uganda and Kenya (Recommendations are for both countries unless otherwise indicated)
1. Non-discrimination	No specific recommendations
2. Availability	*For Uganda only, policy documents should consistently:* 2.1: Prioritise the continuous supply of contraceptive commodities and supplies available through AYF service delivery points
	2.1: Ensure the full financing of family planning commodities in the public and private sectors to prevent stock-outs that affect adolescents
3. Accessibility	3.2: Eliminate financial barriers to contraceptive use by marginalized populations including adolescents and the poor, and make contraceptives affordable to all
	3.8: Ensure mobile access services are used to improve access to contraceptive information and services for adolescents who face geographical barriers to access
	*For Uganda only:* 3.8: Establish different service delivery models to reach adolescents through community based initiatives in non-medical settings (i.e. youth groups, churches, support groups, etc.), clinical settings, school settings, and virtually including through digital platforms
	3.9–3.10: Eliminate third-party authorisation requirements, including spousal and parental authorisation for adolescents accessing contraceptive information and services
4. Acceptability	*For Uganda only:* 4.1: Ensure gender-sensitive counselling and educational interventions on family planning and contraceptives for adolescents
	*For Uganda only:* 4.1: Prioritise programmes for male involvement in the prevention of early and unintended pregnancy among adolescents
	*For Uganda only:* 4.1: Establish community-based service delivery points and distribution of contraception to reach first time parents or young mothers whose mobility is limited, as well as to engage with adolescent couples and fathers in their new parenting roles
	4.2: Provide adolescents with follow-up services for management of contraceptive side-effects as an essential component of all contraceptive service delivery
	4.2: Provide adolescents with referrals for methods not available on site be offered and available
5. Quality	5.1: Ensure quality assurance processes, including medical standards of care and client feedback, be incorporated routinely into contraceptive programmes for adolescents
	5.2: Provide long-acting reversible contraception (LARC) methods to adolescents, including insertion and removal services, and counselling on side-effects, in the same locality
	*For Uganda only:* 5.3: Ensure ongoing competency-based training and supervision of health-care personnel on the delivery of contraceptive education, information and services to adolescents
	*For Uganda only:* 5.3: Strengthen human resources and skills, build capacity on all FP methods, strengthening pre-service training for practice, AYF service approaches, and create internship training for new graduates
6. Informed decision-making	6.1–6.2: Provide evidence-based information about a range of methods for comprehensive, informed choices, and the use of modern contraception without discrimination to adolescents
7. Privacy and confidentiality	7.1: Respect the privacy of adolescents at any service delivery point (AYF or otherwise), particularly regarding contraceptive information and services
8. Participation	8.1: Ensure that communities, particularly adolescents directly affected, have the opportunity to be meaningfully engaged in all aspects of contraceptive programme and policy design, implementation and monitoring for adolescents
9. Accountability	9.1: Establish effective accountability mechanisms that are accessible for adolescents in the delivery of contraceptive information and services, including monitoring and evaluation, and remedies and redress, at the individual and systems levels
	9.1: Ensure that adolescents have easy access to a complaints mechanism or ombudsperson who can help assess and remedy barriers to accessing contraception in a timely way for the individual in question and on a systems level
	9.2: Evaluate and monitor all programmes to guarantee the highest quality of services and respect for human rights particularly for adolescents
	9.2: Include indicators specific to adolescents, including the teenage pregnancy rate and inclusion of women who are unmarried in the calculation of unmet need for contraceptive services
	9.2: In settings where performance-based financing (PBF) occurs, provide a system of checks and balances for adolescents, including assurance of non-coercion and protection of human rights. If PBF occurs, evaluate its effectiveness and its impact on adolescents in terms of increasing availability

Inconsistencies in the content of different adolescent SRH-related laws and policies are a recognised challenge to sound policy implementation in the Sub-Saharan African region [25, 26]. One example from our study illustrates this challenge: The Ugandan *National HIV/AIDS Strategic Plan (2015–2020)* seeks to scale-up comprehensive SRH/HIV programs targeting adolescents (both in and out of school) by providing AYF services including condom use and family planning information and commodities [27]. In 2016, the Ugandan government introduced a parliamentary ban on ‘comprehensive’ sexuality education (beyond abstinence only) [28]. This ban was repealed in 2018 with the launch of the *National Sexuality Education Framework* for adolescents, which does not include condom or contraceptive use [12, 29]. As a result, Uganda has two incoherent policy frameworks in force with regards to the provision of information about contraception to adolescents. Notably, these policies were adopted by different government bodies (Ugandan HIV/AIDS Commission vs. Ministry of Education and Schools), which could further explain the incoherent content. In our study, recommendations for adolescent contraception were predominantly located in policies adopted by the ministries of health or medical services; no such recommendations were identified in policies adopted by the ministries of education (Table 1). There is need therefore to harmonise national laws and policies across sectors for clarity and coherence, which is essential to sound implementation. In addition, a comparison of policy provisions at the national and sub-national levels would be an added value to understand how national framework legislation and policies are formulated, interpreted and applied. Kenya and Uganda are optimal sites for such future research given the decentralisation of health services in 47 counties and 134 districts, respectively. For example, the Makueni County adopted its Maternal and Newborn Child Health Act of 2017, which forbids the sale of condoms to adolescents and children but allows for the sale of any other form of contraception to adolescents who have a high chance of sexual exposure [30]. While our study did not systematically investigate the scope and frequency of policy inconsistencies, they are a major challenge for effective policy implementation and should be further investigated in subsequent research.

This study identified 67 national laws and policies with the potential to address nine domains of adolescent contraception. Of these, only 26 policies had any detailed recommendations for adolescent contraception. This could be the result of public or political opposition to adolescent contraception and a preference for ‘watered-down’ policy statements that do not capture AYF services and information related to contraception. For example, Kenya’s older policies do not include contraception for this reason [31].

This study also illustrates some strengths of Ugandan and Kenyan policy with respect to adolescent contraception (described in the Results). Although a robust policy can create a supportive environment for adolescent contraception, its greatest impact can only be achieved when it is coupled with sound policy implementation. Contraceptive and SRH policy implementation relies on the six building blocks of health systems (including governance, political will, and leadership, adequate financial resources, a skilled and motivated health workforce, reliable and quality service delivery, health information systems, and access to contraceptive commodities), which are persistent challenges in Kenya, Uganda, and other neighbouring countries [25, 31, 32]. However, to the authors’ knowledge, no literature explores how effectively or efficiently the policies in this study were implemented in practice. Yet the consistently low rates of modern contraceptive use among adolescent girls (described below) in Uganda and Kenya suggest that even strong policies are lacking consistent implementation. Drawing on recommendations for policy implementation in other Sub-Sahara African countries, several focal points could also enhance the implementation of Kenyan and Ugandan policies. First, further research on policy implementation that includes ‘robust, pragmatically designed’ evaluation is needed [25]. Monitoring and evaluating the implementation of existing policy frameworks is the first step to addressing barriers to adolescent contraception. Second, implementers are advised to anticipate possible tensions in the policy roll-out (ex. between some health providers’ beliefs or religious communities and policy goals) and proactively develop mitigation strategies [25]. Third, greater cohesion between policy actors, such as donors, governmental ministries or agencies (addressed above), should be sought [25].

Population health indicators illustrate the historical evolution in SRH outcomes in Uganda and Kenya, although no causal relationship between policy changes and outcomes can be determined. The maternal mortality ratio (MMR) has decreased from 468 (2011) to 336 (2016) per 100,000 live births in Uganda and from 520 (2008) to 362 (2014) per 100,000 live births in Kenya. This is partly due to the increased percentage of women using modern methods of family planning from 8 (1995) to 35% (2016) in Uganda and from 39 (2008) to 53% (2014) in Kenya. However, the current use of modern contraceptives among adolescent girls aged 15–19 (who are sexually active and do not want a child in the coming two years) is still low in both countries at 39% in Uganda and 46% in Kenya [33, 34]. Despite the improvements, neither country met the MDG targets on maternal mortality and neither are likely to meet the SDG target 3.1 of reducing MMR to 70/100,000 live births unless adolescent contraception is improved [7, 8]. To make progress towards the SGD targets, policy makers should utilise WHO’s comprehensive list of recommendations to promote adolescent SHR and human rights and ensure their implementation.

The social, political, economic, and public health determinants influencing Uganda and Kenya’s respective policy choices for adolescent contraception have been identified but a political solution has not been reached to address the challenges [29, 31, 35–37]. For example, in both countries, adolescents aged below 18 years are considered children which brings moral questions for age of consent to contraceptive services and therefore the policies remain silent over the issue [12, 15]. Structural barriers in the health system impair service provision such as inadequate physical space in clinics for adolescent contraception ‘corners’, insufficient human resources to staff those corners, and lack of availability of contraceptives affect access to contraceptives for adolescents [29, 31, 35–37]. Future research should investigate the factors that determine the political priority accorded to SRH, and specifically adolescent contraception as a national policy issue. For example, the new Reproductive Healthcare Bill proposed in 2019 in Kenya is a unique opportunity to study political priority for adolescent reproductive health in real-time because it obliges the State to formulate and implement a national strategy and plan of action to realise the right to reproductive health including a specific part on the provision of AYF reproductive health care services [38].

This study’s strengths are its use of primary sources (legal documents in their original language), its consideration of any legal instrument related to adolescent contraception (although not necessarily published by the Ministries of Health), and its use of the WHO recommendations as a standard measure of comparison. WHO’s guidelines were a useful analytical framework for inter-country comparison, including with previous results from South Africa, the Philippines, and Paraguay [16–18]. However, WHO’s guidelines only allow researchers to indicate whether a policy is aligned (or not) with WHO’s best practice, but not whether national policy is regressive. For example, WHO’s recommendations assess whether information on contraceptives is given during or after abortion services, but do not evaluate if legal or accessible safe abortion is a policy priority. The risk of interpretation error was minimised by relying on the definitions of terms and concepts in the policies themselves or, if not provided in the policies, as provided by the MoH. This paper examines national laws and policies adopted between 2010 and 2018, which extends before the 2014 WHO guidelines on ensuring human rights in the provision of contraception were published. On one hand, we can expect that the early laws and policies may not embody WHO’s guidance. On the other hand, WHO guidelines are often based on new initiatives (be it in law or policy) and/or the best practices of several countries. The study does not examine the implementation of policies and their intermediate (such as budget allocation) nor final outcomes (such as better access for adolescents to contraceptives). No traditional stakeholder validation meeting could be held in Kenya due to the travel restrictions and social distancing measures adopted in response to the Coronavirus pandemic.

## Conclusion

Policies in Uganda and Kenya address human rights aspects in the provision of contraceptive information and services to adolescents. However, Kenya’s policies address more of WHO’s recommendations for adolescent contraception than Uganda’s policies. This study highlights strengths, gaps, and areas of incoherence in policies on adolescent contraception in Uganda and Kenya, which are areas of action in future policy frameworks, such as Uganda’s future (national) Adolescent Health Policy and the Reproductive Healthcare Bills that are adopted at the level of Kenyan counties. Aligning national laws and policies with WHO’s recommendations on contraceptive information and services for adolescents may support interventions to improve health outcomes, provided these legal frameworks are effectively implemented.

## Supplementary Information

**Additional file 1.** List of documents screened for inclusion in the article *‘A comparative human rights analysis of laws and policies for adolescent contraception in Uganda and Kenya’*.

## Data Availability

All data analysed during this study were collected from publicly available documents listed in Table 1 and Additional file 1.

## Abbreviations

ART: Antiretroviral therapy
AYF: Adolescent- and youth-friendly
EAC: East African Community
HIV: Human immunodeficiency virus
LARC: Long acting reversible contraceptive
MDG: Millennium development goals
MMR: Maternal mortality rate
MoH: Ministry of Health
PBF: Performance-based financing
SDGs: Sustainable development goals
SRH: Sexual and reproductive health
WHO: World Health Organization
