# Supplementary material for: A comparative human rights analysis of laws and policies for adolescent contraception in Uganda and Kenya
Source: Reprod Health. 2022 Feb 7;19:37. doi: 10.1186/s12978-021-01303-8 (PMC8822716; doi:10.1186/s12978-021-01303-8)
Supplement: Supplementary file 1 — Additional file 1. List of documents screened for inclusion in the article ‘A comparative human rights analysis of laws and policies for adolescent contraception in Uganda and Kenya’. [file 12978_2021_1303_MOESM1_ESM.docx]

**Additional file 1.** List of documents screened for inclusion in the article *‘**A comparative human rights analysis of laws and policies for adolescent contraception in Uganda and Kenya’*.

**Ugandan Documents**

| 1918 | Venereal Diseases Act (Act No. 46) |
| --- | --- |
| 1935 | Public Health Act |
| 1950 | Penal Code Act (Cap. 120) |
| 1971 | Pharmacy and Drugs Act (Cap 280) |
| 1993 | Uganda Family Planning and Maternal Health Policy Guidelines 1993 |
| 2000 | National Health Policy |
| 2000 | National Adolescent Health Policy 2000 |
| 2001 | National  Youth Policy |
| 2003 | Policy for reduction of mother-to-child HIV transmission in Uganda |
| 2003 | Revised National Strategic Framework for HIV/AIDS Activities in Uganda: 2003/04 – 2005/06 |
| 2004 | National Adolescent Health Policy for Uganda |
| 2006 | National Policy Guidelines and Service Standards for Sexual and Reproductive Health and Rights |
| 2007 | Roadmap for Accelerating the Reduction of Maternal and Neonatal Mortality and Morbidity in Uganda 2007-2015 |
| 2007 | National Policy on HIV/AIDS and the World of Work |
| 2007 | National HIV and AIDS Strategic Plan |
| 2007 | Uganda National Gender Policy 2007 |
| 2007 | Roadmap for Accelerating the Reduction of Maternal and Neonatal Mortality and Morbidity in Uganda, 2007– 2015 |
| 2008 | National School Health Policy 2008 |
| 2009 | Reproductive Health Commodity Security Strategic Plan, 2009/10– 2013/14 |
| 2009 | Patients' Charter |
| 2010 | National HIV Testing Services Policy and Implementation Guidelines Uganda |
| 2010 | Uganda HIV Counselling and testing Policy 3rd Edition December, 2010 |
| 2010 | Paediatric HIV Communication Campaign Strategy |
| 2010 | Prohibition of Female Genital Mutilation Act |
| 2010 | Second national health policy |
| 2011 | National HIV Prevention Strategy for Uganda 2011-15 |
| 2011 | National Adolescent Health Strategy 2011-2015 |
| 2011 | NATIONAL STRATEGIC PLAN FOR HIV&AIDS 2011/12 -2014/15 (Revised) |
| 2011 | National Policy Guidelines and Service Standards for Sexual Reproductive Health and Rights of 2011 |
| 2012 | Adolescent Health Policy and Guidelines and Service Standards |
| 2013 | National Condom Programming Strategy |
| 2014 | HIV and AIDs Prevention Control Act, 2014 |
| 2015 | Reducing Morbidity and Mortality from Unsafe abortions in Uganda: Standards and Guidelines |
| 2015 | Uganda Family Planning Cost Implementation Plan 2015-2020 |
| 2015 | National Strategic Plan for HIV/AIDS 2015/16-2019/20 and Priority Action Plan |
| 2015 | Uganda Health Sector Development Plan 2015/16 - 2019/20 |
| 2015 | National School Health Policy 2015 |
| 2016 | National Integrated Early Childhood Development Policy Action Plan of Uganda (2016-2021) |
| 2016 | Uganda Ministry of Health, National Policy Guidelines and Service Standards for Sexual and Reproductive Health and Rights (2016/2018) |
| 2016 | National Youth Policy |
| 2017 | Child and Adolescent Mental Health Policy Guidelines 2017 |
| 2018 | National Sexuality Education Framework |

**Kenyan documents**

| 2000 | National HIV/AIDS Strategic plan 2000-2005 |
| --- | --- |
| 2001 | National Condom Policy and Strategy 2001-2005 |
| 2003 | Adolescent Reproductive Health and Development Policy 2003 |
| 2003 | Education re-entry policy, 2003 |
| 2003 | Persons With Disabilities Act, 2003 |
| 2004 | Policy on HIV in the Education Sector, 2004 |
| 2005 | Adolescent Reproductive Health and Development Policy Plan of Action |
| 2006 | HIV & AIDS Prevention and Control Act, 2006 |
| 2006 | Sexual Offences Act, 2006 |
| 2006 | National Youth Policy, 2006 |
| 2007 | Kenya Nutrition and HIV/AIDS Strategy 2007-2010 |
| 2007 | National Reproductive Health Policy 2007 |
| 2008 | Strategy for Improving the Uptake of Long-acting and Permanent Methods of Contraception in the Family Planning Program 2008-2010 |
| 2008 | Kenya Vision 2030 Midterm Strategic Plan 2 2008-2012 |
| 2008 | Ministry of Medical Services Strategic Plan 2008-2012 |
| 2008 | Ministry of Public Health and Sanitation Strategic Plan 2008-2012 |
| 2008 | Gender Policy in Education, 2008 |
| 2009 | National Reproductive Health Integration Strategy 2009 – 2015 |
| 2009 | National AIDS Strategic Plan 2009/10-2012/13 |
| 2009 | National HIV and AIDS Monitoring, Evaluation and Research Framework (2009/10-2012/13) |
| 2009 | National Condom Policy and Strategy, 2009–2014 |
| 2009 | National School Health Policy, 2009 |
| 2009 | Decentralization Guidelines for HIV/AIDS care and Treatment (2009) |
| 2009 | The National Youth Council Act, No. 10 of 2009 |
| 2010 | National Road Map for Accelerating the Attainment of the MDGs Related to Maternal Health and Newborn Health in Kenya (2010) |
| 2010 | National Family Planning Guidelines for Service |
| 2010 | National Quality management Guidance framework for HIV Testing and Counselling in Kenya (2010) |
| 2010 | Reproductive Health Communication Strategy, Implementation Guide 2010–2012 |
| 2010 | Constitution of Kenya 2010 |
| 2011 | Prohibition of Female Genital Mutilation Act, 2011 |
| 2011 | Gender Policy, 2011 |
| 2011 | Kenya National School Health Strategy, 2011–2015 |
| 2012 | Towards the Elimination of Mother to Child Transmission (eMTCT) of HIV and Keeping Mothers Alive- Strategic Framework 2012 |
| 2012 | National Communication Strategy for Community Health Services, 2012–2017 |
| 2012 | Population Policy for National Development, 2012–2030 |
| 2012 | Kenya HIV Prevention Intervention Assessment Tool |
| 2012 | Public Health Act 1986, revised 2012 |
| 2013 | Kenya Health Sector Strategic and Investment Plan (2013-2017) |
| 2013 | Kenya Vision 2030 Midterm Strategic Plan 2 2013-2017 |
| 2013 | Education Sector Policy on HIV and AIDS, 2013 |
| 2014 | Kenya Health Policy 2014-2030 |
| 2014 | Marriage Act, 2014 |
| 2014 | Kenya HIV Quality Improvement Framework (KHQIF) 2014 |
| 2014 | Reproductive Health Bill 2014 (proposed) |
| 2014 | Adolescents Package of Care in Kenya |
| 2015 | National Adolescent Sexual and Reproductive Health Policy 2015 |
| 2016 | National Guidelines for Provision of Adolescent and Youth Friendly Services in Kenya 2016 |
| 2016 | Children’s Act, 2001, revised 2016 |
| 2017 | National Family Planning Costed Implementation Plan 2017-2020 |
| 2018 | Kenya Vision 2030 Midterm Strategic Plan 3 2018-2022 |
| 2018 | National Youth Service Act, 2018 |
